# Clinical Characteristics, Management, and In-Hospital Outcomes in Patients With Stroke or Transient Ischemic Attack in China

**DOI:** 10.1001/jamanetworkopen.2021.20745

**Published:** 2021-08-13

**Authors:** Hong-Qiu Gu, Xin Yang, Chun-Juan Wang, Xing-Quan Zhao, Yi-Long Wang, Li-Ping Liu, Xia Meng, Yong Jiang, Hao Li, Chelsea Liu, Runqi Wangqin, Gregg C. Fonarow, Lee H. Schwamm, Ying Xian, Zi-Xiao Li, Yong-Jun Wang

**Affiliations:** 1 China National Clinical Research Center for Neurological Diseases, Beijing Tiantan Hospital, Capital Medical University, Beijing, China; 2 National Center for Healthcare Quality Management in Neurological Diseases, Beijing Tiantan Hospital, Capital Medical University, Beijing, China; 3 Vascular Neurology, Department of Neurology, Beijing Tiantan Hospital, Capital Medical University, Beijing, China; 4 Neuro-intensive Care Unit, Department of Neurology, Beijing Tiantan Hospital, Capital Medical University, Beijing, China; 5 Department of Epidemiology, Harvard T. H. Chan School of Public Health, Boston, Massachusetts; 6 Department of Neurology, Duke University Medical Center, Durham, North Carolina; 7 Ahmanson-UCLA Cardiomyopathy Center, Ronald Reagan–UCLA Medical Center, Los Angeles, California; 8 Associate Editor for Health Care Quality and Guidelines, *JAMA Cardiology*; 9 Department of Neurology, Massachusetts General Hospital, Harvard Medical School, Boston; 10 Chinese Institute for Brain Research, Beijing, China

## Abstract

**Importance:**

Stroke represents a significant burden on the health care system of China. The Chinese Stroke Center Alliance was launched in 2015 to monitor and improve care quality and outcomes for patients with acute stroke and transient ischemic attack (TIA).

**Objective:**

To evaluate the clinical characteristics, management, and in-hospital clinical outcomes and complications among patients with stroke or TIA in China.

**Design, Setting, and Participants:**

This quality improvement study assessed stroke or TIA admissions to 1476 participating hospitals in the Chinese Stroke Center Alliance between August 1, 2015, and July 31, 2019.

**Exposures:**

Stroke types and calendar year.

**Main Outcomes and Measures:**

Eleven guideline-based admission or discharge management measures and 2 summary measures: an all-or-none binary outcome and a composite score (range, 0 [nonadherence] to 1 [perfect adherence]) for adherence to evidence-based stroke and TIA care and in-hospital clinical outcomes, including death or discharge against medical advice (DAMA), major adverse cardiovascular events (MACEs), including ischemic stroke, hemorrhagic stroke, TIA, or myocardial infarction; and in-hospital complications.

**Results:**

Of 1 006 798 patients with stroke or TIA (mean [SD] age, 65.7 [12.2] years; 383 500 [38.1%] female), 838 229 (83.3%) had an ischemic stroke, 64 929 (6.4%) had TIA, 85 705 (8.5%) had intracerebral hemorrhage (ICH), and 11 241 (1.1%) had subarachnoid hemorrhage (SAH). Management measures varied by cerebrovascular event type, with the mean (SD) composite score ranging from 0.57 (0.31) in SAH to 0.83 (0.24) in TIA. Poor outcomes and complications were highest among patients with SAH (21.9%; 95% CI, 21.0%-22.8% in-hospital death or DAMA; 9.6%; 95% CI, 9.1%-10.2% MACEs; and 31.4%; 95% CI, 30.6%-32.3% in-hospital complications) and patients with ICH (17.2%; 95% CI, 16.9%-17.5% in-hospital death or DAMA; 9.3%; 95% CI, 9.1%-9.5% MACEs; and 31.3%; 95% CI, 31.0%-31.6% in-hospital complications), followed by patients with ischemic stroke (6.1%; 95% CI, 6.0%-6.1% in-hospital death or DAMA; 6.3%; 95% CI, 6.3%-6.4% MACEs; and 12.8%; 95% CI, 12.7%-12.9% in-hospital complications), and lowest in patients with TIA (5.0%; 95% CI, 4.8%-5.2% in-hospital death or DAMA; 2.4%; 95% CI, 2.3%-2.5% MACEs; and 0.8%; 95% CI, 0.7%-0.8% in-hospital complications). Temporal improvements in management measures were observed from 2015 to 2019, especially in administration of intravenous recombinant tissue plasminogen activator (+60.3% relatively; 95% CI, 52.9%-70.5%), dysphagia screening (+14.7% relatively; 95% CI, 14.0%-15.6%), and use of anticoagulants for atrial fibrillation (+31.4% relatively; 95% CI, 25.7%-37.3%). Temporal improvements in in-hospital death or DAMA (−9.7% relatively; 95% CI, −9.6% to −8.5%) and complications (−27.1% relatively; 95% CI, −28.6% to −25.3) were also observed.

**Conclusions and Relevance:**

In this quality improvement study, performance measure adherence and poor outcomes and complications varied by cerebrovascular event type; although there were substantial improvements over time, these results suggest that support for the use of evidence-based practices is needed.

## Key Points

**Question:**

What are the contemporary clinical characteristics, management, and in-hospital outcomes in patients with acute stroke and transient ischemic attack in China?

**Findings:**

In this quality improvement study that included more than 1 million admissions, in-hospital management measures and outcomes varied by type of cerebrovascular event and hospital level of care. Temporal improvements from 2015 to 2019 were also observed.

**Meaning:**

Although improvements were seen over time, these findings suggest that ongoing support for evidence-based care is needed.

## Teaser

This quality improvement study examines variations in and temporal trends of performance measures and in-hospital outcomes in patients with stroke or transient ischemic attack in the Chinese Stroke Center Alliance program.

## Introduction

Stroke is the leading cause of death and adult disability in China,^1,2^ with more than 13 million strokes and another 23.9 million transient ischemic attacks (TIAs) each year.^3,4^ Despite evidence-based treatments and guidelines for stroke and TIA,^5,6,7^ variation still exists in adherence to evidence-based stroke and TIA care.^8,9^ A multifaceted quality improvement intervention was developed and implemented to improve adherence to evidence-based performance measures and in-hospital outcomes in patients with acute ischemic stroke (IS) in China.^10^ Furthermore, several organizations in China have developed registries to measure, track, and improve acute stroke care.^8,11,12^

Prior studies^8,13,14^ have described the quality of care and outcomes for patients hospitalized with stroke or TIA in China, but they have been largely limited to hospitals in urban regions or in a small subset of provinces. The Chinese Stroke Center Alliance (CSCA) was developed by the Chinese Stroke Association as a national effort to improve health care quality for acute stroke and TIA as well as to generate data for stroke research.^15,16^ Given its national scope, duration, and prospective data collection methods, the CSCA provides a unique opportunity to better understand a large cohort of patients hospitalized with stroke and TIA in China. In the current analysis, we aimed to characterize the first 1 million hospitalizations and to examine variations and temporal trends in adherence to guideline-based performance measures and in-hospital outcomes in the CSCA program.

## Methods

### The CSCA

The CSCA is a national, hospital-based, voluntary, and continuous quality improvement initiative that provides a unique platform to develop stroke centers and improve stroke care quality and clinical outcomes modeled after the American Heart Association’s Get With the Guidelines–Stroke (GWTG-Stroke) program.^17^ This program was made available to all secondary and tertiary hospitals in China. More details on types of participating hospitals and their representativeness in China are available in the eMethods and eFigure 1 in the Supplement. Hospitals joined the program in a staggered manner. The protocol for case identification and data collection has been previously reported elsewhere.^15^ Patients were enrolled if they had a primary diagnosis of stroke or TIA that was confirmed by brain computed tomography or magnetic resonance imaging. Data were collected by trained hospital personnel using a web-based electronic case report form (Medicine Innovation Research Center, Beijing, China). The CSCA program was approved by the ethics committee of Beijing Tiantan Hospital. Each participating hospital received institutional review board approval to enroll participants without individual patient consent under the Common Rule or a waiver of authorization and exemption. All the data were deidentified and collected from routine clinical practice. The collection process did not change the routine clinical practice. This report followed the Standards for Quality Improvement Reporting Excellence (SQUIRE) reporting guideline.

### Multifaceted Quality Improvement Initiative

The multifaceted quality improvement initiative includes 2 parts, which were developed under the concept of the Plan-Do-Check-Act cycle. The first part is the web-based data collection and feedback system,^18^ which collects concurrent data via predefined logic features, range checks, and user alerts and provides feedback for key performance measures proven by a randomized clinical trial of multifaceted quality improvement intervention^10^ to allow participating hospitals to compare their performance to the past and current standards of other regional hospitals. The second part is the construction of stroke centers through collaborative workshops and webinars given by China Stroke Association staff and volunteers with expertise in clinical science and quality improvement; workshops and webinars included didactic presentation of clinical trial evidence, guideline-recommended performance measures for acute stroke management and secondary prevention of stroke and TIA, and examples of successful hospital implementation. Videos of these workshops were readily available online and via mobile apps. More details can be found in the CSCA protocol.^19^

### Study Population

The population for this study consisted of all patients with stroke or TIA from secondary or tertiary hospitals in the CSCA program across 31 provinces, autonomous regions, and municipalities in mainland China (eTable 1 in the Supplement). A total of 1 015 510 patients with stroke or TIA were enrolled between August 1, 2015, and July 31, 2019, from 1476 participating hospitals. We excluded 8712 cases (0.9%) with missing data on stroke type, age, sex, insurance, medical history, or hospital level. The final analytic cohort consisted of 1 006 798 stroke or TIA admissions (eFigure 2 in the Supplement). Enrollment in CSCA increased progressively, with 60 000 to 80 000 participants enrolled per quarter from August 1, 2015, to June 30, 2018 (eFigure 3 in the Supplement).

### In-Hospital Management Measures

A total of 11 guideline-recommended performance measures for in-hospital management were prespecified for this study based on national recommended guidelines and the GWTG-Stroke criteria.^20^ These measures included 5 admission performance measures and 6 discharge performance measures. The 5 admission performance measures included (1) intravenous recombinant tissue plasminogen activator (IV-rtPA) in patients who arrived within 3.5 hours after symptom onset and were treated within 4.5 hours, (2) antithrombotic medication administration within 48 hours of admission, (3) deep vein thrombosis (DVT) prophylaxis, (4) dysphagia screening, and (5) rehabilitation assessment. The 6 discharge performance measures included (1) antithrombotic medication, (2) anticoagulants for atrial fibrillation, (3) antihypertensive medication for patients with hypertension, (4) hypoglycemia medication for diabetes, (5) lipid-lowering medications in patients with low-density lipoprotein cholesterol (LDL-C) levels of 100 mg/dL or higher or not documented (to convert to millimoles per liter, multiply by 0.0259), and (6) smoking cessation intervention. The detailed definitions of these performance measures are given in eTable 2 in the Supplement. In addition, 2 summary measures were defined. An all-or-none binary variable defined whether patients received all the performance measure interventions for which they were eligible. A composite measure of care (range, 0 [non-adherence] to 1 [perfect adherence]) for adherence to evidence-based stroke or TIA care was defined as the number of performance measures actually performed divided by the total number of eligible performance measures for a patient. For subarachnoid hemorrhage (SAH), intracranial hemorrhage (ICH), and strokes of undetermined origin, the composite measures were calculated based on only the applicable performance measures for each cerebrovascular event type.

### In-Hospital Outcomes

In-hospital outcomes assessed in this study included death or discharge against medical advice (DAMA), major adverse cardiovascular events (MACEs) (IS, hemorrhagic stroke, TIA, or myocardial infarction [MI]), complications (DVT, pneumonia, pulmonary embolism, epileptic seizure, hydrocephalus, urinary tract infection, respiratory failure or cardiopulmonary arrest, bedsore [decubitus ulcer], depression, or gastrointestinal bleeding), and length of stay in the hospital.

### Statistical Analysis

We described the patient or hospital characteristics by cerebrovascular event types. Categorical variables were reported as numbers (percentages), and continuous variables were reported as means (SDs) or medians (interquartile ranges [IQRs]). We did not perform statistical tests for the comparison of different cerebrovascular event types because statistical significance attributable to the large sample size may not indicate clinical significance. Cochran-Armitage trend tests were used to assess the linear trends in performance measures and clinical outcomes from 2015 to 2019. To account for within-hospital clustering and covariates, we also used logistic regression models with generalized estimating equations to assess the temporal trends in quality of care and in-hospital outcomes, treating patients in the same hospital as a cluster in the model and adjusting for patient characteristics (age, sex, smoking, and insurance status), stroke severity measured by the National Institutes of Health Stroke Scale (NIHSS) score, medical history (prior stroke or TIA, hypertension, diabetes, dyslipidemia, coronary heart disease [CHD] or MI, atrial fibrillation or flutter, heart failure, and peripheral vascular disease), and hospital characteristics (hospital level and region). We also reported median (IQR) for each performance measure and in-hospital outcome at the hospital level to show variations across hospitals.

All statistical analyses were performed using SAS software, version 9.4 (SAS Institute Inc). Descriptive tables were produced by an SAS macro (%ggBaseline) that can automatically generate baseline tables.^21^ All P values are 2-sided, with *P* < .05 considered significant.

## Results

A total of 1 006 798 patients (mean [SD] age, 65.7 [12.2] years; 383 500 [38.1%] female) with stroke or TIA from 1476 hospitals were included in this analysis. A total of 838 229 patients (83.3%) had ISs, 64 929 (6.4%) had TIAs, 85 705 (8.5%) had ICHs, 11 241 (1.1%) had SAHs, and 6694 (0.7%) had strokes of undetermined origin.

Table 1 summarizes the demographic and clinical characteristics by cerebrovascular event type. Participants with ISs had a relatively high prevalence of atrial fibrillation or flutter (44 802 [5.3%]), prior CHD or MI (75 466 [9.0%]), and diabetes (179 794 [21.4%]) compared with participants with other stroke types; participants with ICHs had a higher prevalence of hypertension (61 128 [71.3%]); and participants with SAHs were younger (mean [SD] age, 60.0 [12.9] years) and more likely to be women (6643 [59.1%]).

**Table 1.** Baseline Characteristics of the Chinese Stroke Center Alliance Population by Cerebrovascular Event Type

Characteristic	No. (%) of patients					
	Total (n = 1 006 798 [100%])	IS (n = 838 229 [83.3%])	TIA (n = 64 929 [6.4%])	ICH (n = 85 705 [8.5%])	SAH (n = 11 241 [1.1%])	SNC (n = 6694 [0.7%])
Demographic characteristics						
Age, y						
Mean (SD)	65.7 (12.2)	66.2 (12.0)	64.3 (12.2)	62.9 (12.9)	60.0 (12.9)	64.4 (13.6)
Median (IQR)	66.0 (57.0-75.0)	67.0 (58.0-75.0)	65.0 (55.0-73.0)	63.0 (53.0-72.0)	60.0 (51.0-69.0)	65.0 (55.0-74.0)
Sex						
Male	623 298 (61.9)	524 351 (62.6)	37 351 (57.5)	53 543 (62.5)	4598 (40.9)	3455 (51.6)
Female	383 500 (38.1)	313 878 (37.4)	27 578 (42.5)	32 162 (37.5)	6643 (59.1)	3239 (48.4)
Current or history of smoking	363 797 (36.1)	310 811 (37.1)	21 137 (32.6)	27 865 (32.5)	2416 (21.5)	1568 (23.4)
Insurance						
UEBMI	283 778 (28.2)	240 261 (28.7)	22 615 (34.8)	17 056 (19.9)	1946 (17.3)	1900 (28.4)
URBMI	185 950 (18.5)	158 383 (18.9)	11 335 (17.5)	13 454 (15.7)	1724 (15.3)	1054 (15.7)
NRCMS	430 935 (42.8)	351 637 (41.9)	25 366 (39.1)	45 251 (52.8)	5969 (53.1)	2712 (40.5)
Self-pay	64 519 (6.4)	53 365 (6.4)	3068 (4.7)	6691 (7.8)	1037 (9.2)	358 (5.3)
Other	41 616 (4.1)	34 583 (4.1)	2545 (3.9)	3253 (3.8)	565 (5.0)	670 (10.0)
In-hospital NIHSS score						
Undocumented	264 272 (26.2)	169 991 (20.3)	47 897 (73.8)	36 443 (42.5)	6336 (56.4)	3605 (53.9)
0-4	472 068 (46.9)	429 564 (51.2)	15 583 (24.0)	21 348 (24.9)	3252 (28.9)	2321 (34.7)
5-14	217 556 (21.6)	196 594 (23.5)	1193 (1.8)	18 400 (21.5)	827 (7.4)	542 (8.1)
≥15	52 902 (5.3)	42 080 (5.0)	256 (0.4)	9514 (11.1)	826 (7.3)	226 (3.4)
Medical history						
Prior stroke or TIA	329 278 (32.7)	279 159 (33.3)	21 740 (33.5)	24 622 (28.7)	2340 (20.8)	1417 (21.2)
Hypertension	646 920 (64.3)	539 638 (64.4)	37 241 (57.4)	61 128 (71.3)	5585 (49.7)	3328 (49.7)
Diabetes	200 803 (19.9)	179 794 (21.4)	11 051 (17.0)	8146 (9.5)	750 (6.7)	1062 (15.9)
Dyslipidemia	74 150 (7.4)	64 600 (7.7)	5189 (8.0)	3644 (4.3)	350 (3.1)	367 (5.5)
Prior CHD or MI	87 579 (8.7)	75 466 (9.0)	6180 (9.5)	4818 (5.6)	565 (5.0)	550 (8.2)
Atrial fibrillation or flutter	48 271 (4.8)	44 802 (5.3)	1789 (2.8)	1312 (1.5)	111 (1.0)	257 (3.8)
Hospital characteristics						
Hospital level						
Secondary	391 714 (38.9)	321 221 (38.3)	27 814 (42.8)	35 689 (41.6)	3512 (31.2)	3478 (52.0)
Tertiary	615 084 (61.1)	517 008 (61.7)	37 115 (57.2)	50 016 (58.4)	7729 (68.8)	3216 (48.0)
Region						
Eastern	451 032 (44.8)	385 913 (46.0)	26 611 (41.0)	31 694 (37.0)	3983 (35.4)	2831 (42.3)
Central	344 146 (34.2)	277 269 (33.1)	25 333 (39.0)	34 385 (40.1)	4717 (42.0)	2442 (36.5)
Western	211 620 (21.0)	175 047 (20.9)	12 985 (20.0)	19 626 (22.9)	2541 (22.6)	1421 (21.2)

Abbreviations: CHD, coronary heart disease; ICH, intracerebral hemorrhage; IQR, interquartile range; IS, ischemic stroke; MI, myocardial infarction; NIHSS, National Institutes of Health Stroke Scale; NRCMS, new rural cooperative medical scheme; PVD, peripheral vascular disease; SAH, subarachnoid hemorrhage; SNC, stroke not classified; TIA, transient ischemic attack; UEBMI, urban employee basic medical insurance; URBMI, urban resident basic medical insurance.

### Health Quality Measures

Adherence to quality measures is detailed in Table 2. The composite score ranged from 0.57 (0.31) in patients with SAH to 0.83 (0.24) in patients with TIA, and the all-or-none measure ranged from 21.2% (95% CI, 20.4%-21.9%) in patients with SAH to 55.8% (95% CI, 55.4%-56.2%) in patients with TIA. Among measures that were applicable to all stroke types, the proportions of those who were assessed for or had received dysphagia screening (78.0%; 95% CI, 77.9%-78.1%), had undergone rehabilitation (73.5%; 95% CI, 73.4%-73.6%), and were administered glucose-lowering medication for hyperglycemia (78.6%; 95% CI, 78.5%-78.8%) were higher among patients with IS, whereas the proportion of those who were administered antihypertensives was higher among patients with ICH (83.8%; 95% CI, 83.5%-84.0%). Use of a smoking cessation intervention was optimal (ie, >90%) across all cerebrovascular event types. Quality measures that were specific to IS or TIA had adherence rates of approximately 85% for early antithrombotics, 88% for antithrombotics at discharge, 42% for anticoagulants to treat atrial fibrillation, and 90% for statin for those with LDL-C levels of 100 mg/dL or higher or not documented. The largest gap in performance measure among cerebrovascular event types was seen in DVT prophylaxis, ranging from 6.2% in patients with strokes of undetermined origin to 24.2% in patient with ICHs.

**Table 2.** Individual Performance Measures and Summary Measures by Cerebrovascular Event Type

Variable	IS (n = 838 229 [83.3%])		TIA (n = 64 929 [6.4%])		ICH (n = 85 705 [8.5%])		SAH (n = 11 241 [1.1%])		SNC (n = 6694 [0.7%])	
	No./total No.	Frequency, % (95% CI)	No./total No.	Frequency, % (95% CI)	No./total No.	Frequency, % (95% CI)	No./total No.	Frequency, % (95% CI)	No./total No.	Frequency, % (95% CI)
Acute performance measures										
IV-rtPA administration ≤4.5 h	43 749/191 389	22.9 (22.7-23.1)	NA	NA	NA	NA	NA	NA	NA	NA
Early antithrombotics	702 177/822 296	85.4 (85.3-85.5)	54 701/63 826	85.7 (85.4-86.0)	NA	NA	NA	NA	NA	NA
DVT prophylaxis	44 524/260 578	17.1 (16.9-17.2)	NA	NA	12 408/51 337	24.2 (23.8-24.5)	1556/6504	23.9 (22.9-25.0)	119/1924	6.2 (5.1-7.3)
Dysphagia screen	653 804/838 229	78.0 (77.9-78.1)	NA	NA	61 334/85 705	71.6 (71.3-71.9)	7092/11 241	63.1 (62.2-64.0)	3859/6694	57.7 (56.5-58.8)
Rehabilitation	615 991/838 229	73.5 (73.4-73.6)	NA	NA	62 228/85 705	72.6 (72.3-72.9)	6513/11 241	57.9 (57.0-58.9)	4426/6694	66.1 (65.0-67.3)
Discharge performance measures										
Antithrombotics	717 843/812 839	88.3 (88.2-88.4)	56 076/63 783	87.9 (87.7-88.2)	NA	NA	NA	NA	NA	NA
Anticoagulants for AF	24 774/59 323	41.8 (41.4-42.2)	906/2385	38.0 (36.0-39.9)	NA	NA	NA	NA	NA	NA
BP lowering for hypertension	403 734/629 678	64.1 (64.0-64.2)	27 519/42 595	64.6 (64.2-65.1)	60 633/72 380	83.8 (83.5-84.0)	4615/6689	69.0 (67.9-70.1)	2543/3820	66.6 (65.1-68.1)
Glucose lowering for hyperglycemia	180 547/229 650	78.6 (78.5-78.8)	10 721/13 828	77.5 (76.8-78.2)	7596/10 974	69.2 (68.4-70.1)	598/1033	57.9 (54.9-60.9)	915/1324	69.1 (66.6-71.6)
Statin for LDL-C ≥100 mg/dL or ND	744 714/828 591	89.9 (89.8-89.9)	57 899/64 538	89.7 (89.5-90.0)	NA	NA	NA	NA	NA	NA
Smoking cessation intervention	191 588/199 195	96.2 (96.1-96.3)	12 978/13 482	96.3 (95.9-96.6)	15 572/16 535	94.2 (93.8-94.5)	1502/1620	92.7 (91.5-94.0)	772/808	95.5 (94.1-97.0)
Summary measures										
Composite score, mean (SD)	0.76 (0.21)		0.83 (0.24)		0.68 (0.28)		0.57 (0.31)		0.61 (0.30)	
All-or-none binary variable	197 768/838 229	23.6 (23.5-23.7)	36 217/64 929	55.8 (55.4-56.2)	24 242/85 705	28.3 (28.0-28.6)	2381/11 241	21.2 (20.4-21.9)	1560/6694	23.3 (22.3-24.3)

Abbreviations: AF, atrial fibrillation; BP, blood pressure; DVT, deep vein thrombosis; ICH, intracerebral hemorrhage; IS, ischemic stroke; IV-rtPA, intravenous recombinant tissue plasminogen activator; LDL-C, low-density lipoprotein cholesterol; NA, not applicable; ND, not documented; SAH, subarachnoid hemorrhage; SNC, stroke not classified; TIA, transient ischemic attack.

SI conversion factor: To convert LDL-C to millimoles per liter, multiply by 0.0259.

### In-Hospital Outcomes

We observed variations by cerebrovascular event types in in-hospital death or DAMA, MACEs, length of stay, and complications (Table 3). In-hospital death or DAMA ranged from 5.0% (95% CI, 4.8%-5.2%) in TIAs to 17.2% (95% CI, 16.9%-17.5%) in ICHs and 21.9% (95% CI, 21.0%-22.8%) in SAHs. Compared with patients with IS, patients with hemorrhagic stroke had a higher rate of MACEs (9.3% [95% CI, 9.1%–9.5%] for ICH, 9.6% [95% CI, 9.1%–10.2%] for SAH, and 6.3% [95% CI, 6.3%–6.4%] for IS) and longer length of stay (median [IQR] days: 15 [10-21] for ICH, 14 [6-20] for SAH, and 11 [8-14] for IS). We also found that patients with hemorrhagic stroke had much higher rates of in-hospital complications compared with patients with IS (31.3% for ICH, 31.4% for SAH, and 12.8% for IS), especially for in-hospital pneumonia (25.5% in ICH, 23.7% in SAH, and 9.2% in IS).

**Table 3.** Stroke-Related In-Hospital Outcomes and Complications by Cerebrovascular Event Type^a^

Variable	IS (n = 838 229 [83.3%])	TIA (n = 64 929 [6.4%])	ICH (n = 85 705 [8.5%])	SAH (n = 11 241 [1.1%])	SNC (n = 6694 [0.7%])
In-hospital death or DAMA^b^	39 453 (6.1) [6.0-6.1]	2397 (5.0) [4.8-5.2]	10 704 (17.2) [16.9-17.5]	1833 (21.9) [21.0-22.8]	308 (6.0) [5.3-6.6]
In-hospital death	3766 (0.5) [0.4-0.5]	21 (0) [0-0.1]	2017 (2.4) [2.3-2.5]	341 (3.0) [2.7-3.4]	33 (0.5) [0.3-0.7]
DAMA^b^	35 687 (5.5) [5.5-5.6]	2376 (5.0) [4.8-5.1]	8687 (14.4) [14.1-14.7]	1492 (18.6) [17.8-19.5]	275 (5.4) [4.8-6.0]
In-hospital MACEs	53 168 (6.3) [6.3-6.4]	1537 (2.4) [2.3-2.5]	8000 (9.3) [9.1-9.5]	1082 (9.6) [9.1-10.2]	333 (5.0) [4.5-5.5]
Cerebral infarction	41 872 (5.0) [5.0-5.0]	197 (0.3) [0.3-0.4]	1083 (1.3) [1.2-1.3]	311 (2.8) [2.5-3.1]	134 (2.0) [1.7-2.3]
Cerebral hemorrhage	7717 (0.9) [0.9-0.9]	33 (0.1) [0.0-0.1]	7074 (8.3) [8.1-8.4]	800 (7.1) [6.6-7.6]	119 (1.8) [1.5-2.1]
TIA	6155 (0.7) [0.7-0.8]	1414 (2.2) [2.1-2.3]	236 (0.3) [0.2-0.3]	56 (0.5) [0.4-0.6]	111 (1.7) [1.4-2.0]
Myocardial infarction	3447 (0.4) [0.4-0.4]	24 (0.0) [0.0-0.1]	215 (0.3) [0.2-0.3]	51 (0.5) [0.3-0.6]	20 (0.3) [0.2-0.4]
Length of stay, median (IQR), d	11 (8-14)	8 (6-10)	15 (10-21)	14 (6-20)	8 (6-12)
In-hospital complications	107 421 (12.8) [12.7-12.9]	490 (0.8) [0.7-0.8]	26 791 (31.3) [31.0-31.6]	3531 (31.4) [30.6-32.3]	694 (10.4) [9.6-11.1]
DVT	7698 (0.9) [0.9-0.9]	52 (0.1) [0.1-0.1]	1126 (1.3) [1.2-1.4]	173 (1.5) [1.3-1.8]	63 (0.9) [0.7-1.2]
Pneumonia	76 745 (9.2) [9.1-9.2]	281 (0.4) [0.4-0.5]	21 874 (25.5) [25.2-25.8]	2658 (23.7) [22.9-24.4]	409 (6.1) [5.5-6.7]
Pulmonary embolism	1736 (0.2) [0.2-0.2]	22 (0.0) [0.0-0.1]	233 (0.3) [0.2-0.3]	45 (0.4) [0.3-0.5]	24 (0.4) [0.2-0.5]
Epileptic seizure	4698 (0.6) [0.5-0.6]	43 (0.1) [0.1-0.1]	1203 (1.4) [1.3-1.5]	228 (2.0) [1.8-2.3]	71 (1.1) [0.8-1.3]
Hydrocephalus	1534 (0.2) [0.2-0.2]	25 (0.0) [0.0-0.1]	1868 (2.2) [2.1-2.3]	537 (4.8) [4.4-5.2]	20 (0.3) [0.2-0.4]
Urinary tract infection	10 710 (1.3) [1.3-1.3]	74 (0.1) [0.1-0.1]	2112 (2.5) [2.4-2.6]	284 (2.5) [2.2-2.8]	90 (1.3) [1.1-1.6]
Respiratory failure or cardiopulmonary arrest	3025 (0.4) [0.4-0.4]	8 (0.0) [0.0-0.0]	1668 (2.0) [1.9-2.0]	375 (3.3) [3.0-3.7]	28 (0.4) [0.3-0.6]
Bedsore	2715 (0.3) [0.3-0.3]	11 (0.0) [0.0-0.0]	530 (0.6) [0.6-0.7]	59 (0.5) [0.4-0.7]	26 (0.4) [0.2-0.5]
Depression	11 300 (1.4) [1.3-1.4]	67 (0.1) [0.1-0.1]	1018 (1.2) [1.1-1.3]	90 (0.8) [0.6-1.0]	70 (1.1) [0.8-1.3]
Gastrointestinal bleeding	7917 (0.9) [0.9-1.0]	30 (0.1) [0.0-0.1]	2458 (2.9) [2.8-3.0]	303 (2.7) [2.4-3.0]	49 (0.7) [0.5-0.9]

Abbreviations: DAMA, discharge against medical advice; DVT, deep vein thrombosis; ICH, intracerebral hemorrhage; IQR, interquartile range; IS, ischemic stroke; MACE, major adverse cardiovascular event; SAH, subarachnoid hemorrhage; SNC, stroke not classified; TIA, transient ischemic attack.

^a^ Data are presented as number (percentage [95% CI]) of events unless otherwise indicated.

^b^ Data on DAMA were missing from 188 619 patients for IS, 16 868 for TIA, 23 427 for ICH, 2878 for SAH, and 1541 for SNC. Discharge against medical advice was assessed among discharged surviving patients.

### Temporal Trend Analysis

**Figure 1.**
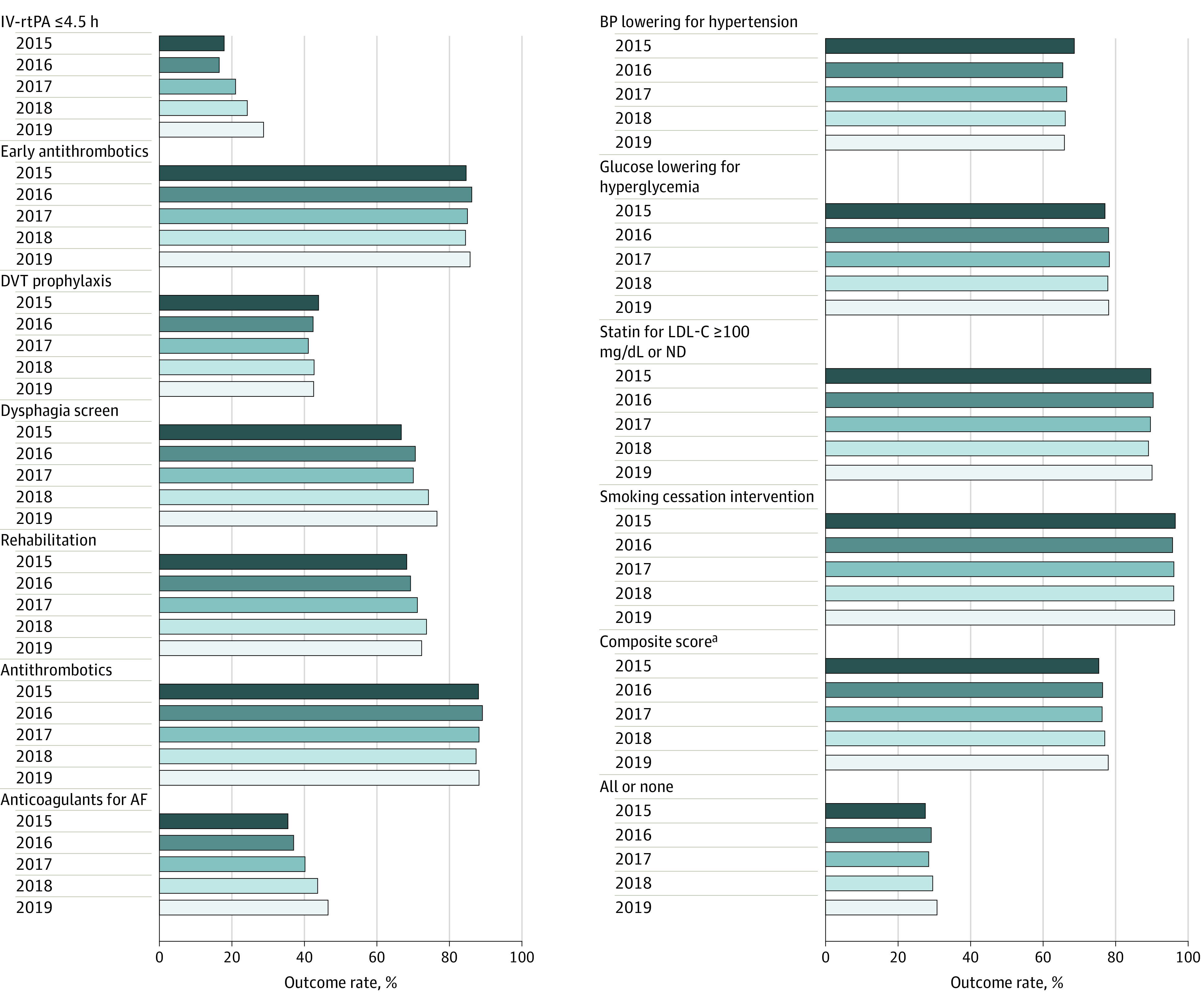
Temporal Trend Analysis of Quality Measures AF indicates atrial fibrillation; BP, blood pressure; DVT, deep vein thrombosis; IV-rtPA, intravenous recombinant tissue plasminogen activator; LDL-C, low-density lipoprotein cholesterol (to convert to millimoles per liter, multiply by 0.0259); and ND, not documented. ^a^The composite score is a continuous outcome ranging from 0 to 1; it is presented on the 0% to 100% scale to show it alongside the other measures in in the figure.

**Figure 2.**
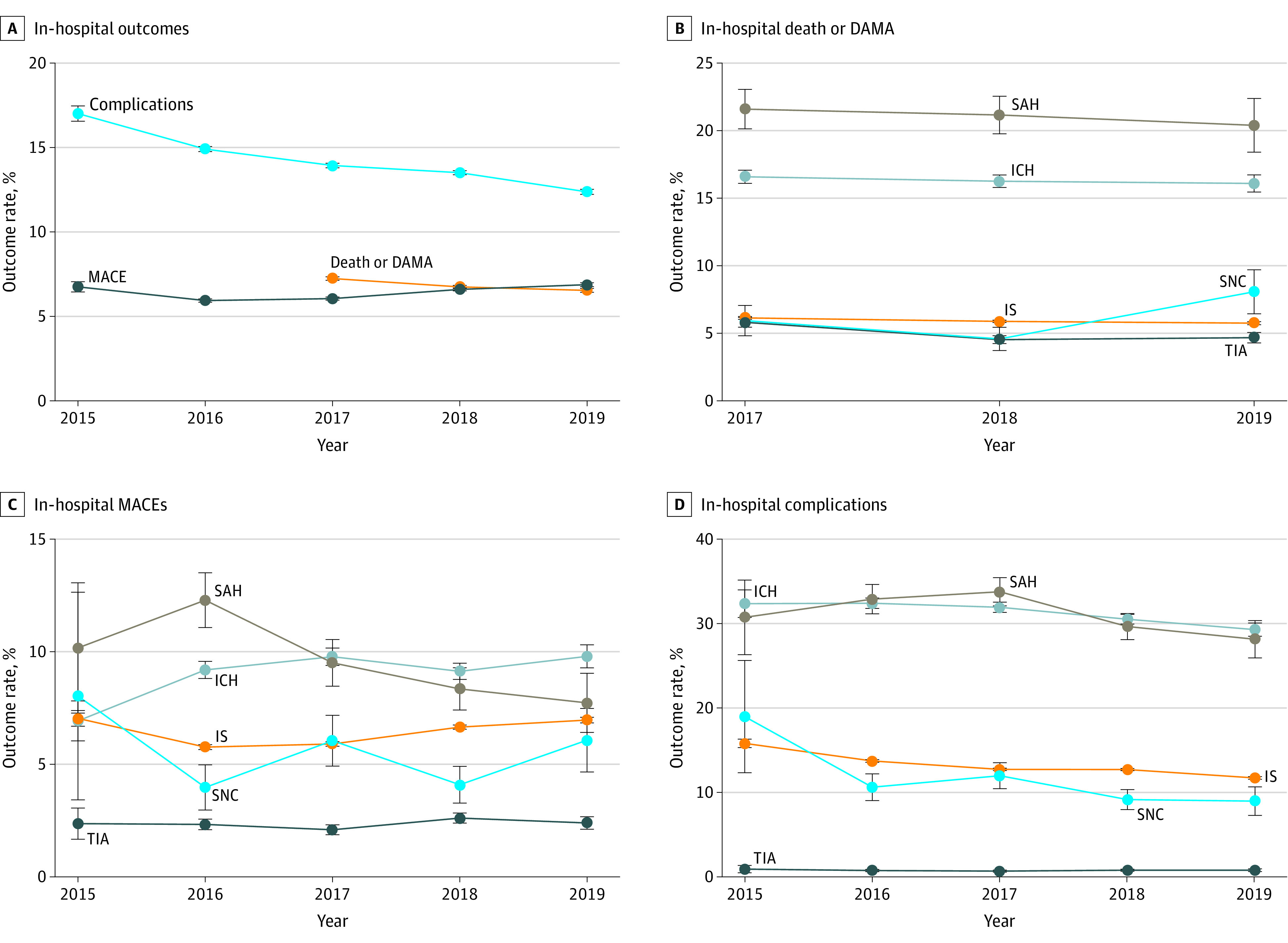
Temporal Trend Analysis of In-Hospital Outcomes Because discharge against medical advice (DAMA) was not a required field in the system before 2017, more than 90% of these data were missing. Thus, the trend analyses were assessed starting from 2017 for in-hospital death and DAMA. Error bars indicate 95% CIs. ICH indicates intracerebral hemorrhage; IS, ischemic stroke; MACE, major adverse cardiovascular event; SAH, subarachnoid hemorrhage; SNC, stroke not classified; and TIA, transient ischemic attack.

From 2015 to 2019, clinically meaningful and statistically significant improvements were found in some measures. Specifically, the rate of IV-rtPA increased by 60.3% (95% CI, 52.9%-70.5%), the rate of dysphagia screening increased by 14.7% (95% CI, 14.0%-15.6%), and administering anticoagulant for atrial fibrillation or flutter increased by 31.4% (95% CI, 25.7%-37.3%) (Figure 1; eTable 3 in the Supplement). We also assessed temporal trends for in-hospital outcomes, and a marked decrease was observed for in-hospital death or DAMA (decreased by 9.7% [95% CI, −9.6% to −8.5%] relatively) and in-hospital complications (decreased by 27.1% [95% CI, −28.6% to −25.3%] relatively). However, decreasing trends were not observed for MACEs, except for SAH and stroke not classified (Figure 2; eTable 4 in the Supplement).

### Variations of In-Hospital Management and Outcomes

We found variations of in-hospital management measures at the hospital level, especially for IV-rtPA administration for 4.5 hours or less (median, 16.9%; IQR, 5.6%-33.3%), DVT prophylaxis (median, 7.7%; IQR, 1.9%-20.9%), and anticoagulants for atrial fibrillation (median, 37.5%; IQR, 22.6%-52.5%) for ISs. Variations of rehabilitation and glucose lowering for hyperglycemia for ICH (rehabilitation: median, 76.3%; IQR, 55.6%-92.6%; glucose lowering for hyperglycemia: median, 75.0%; IQR, 52.0%-100.0%) and SAH (rehabilitation: 55.6%; IQR, 16.7%-100.0%; glucose lowering for hyperglycemia: median, 66.7%; IQR, 0.0%-100.0%) are also notable (eTable 5 in the Supplement). Variations of in-hospital outcomes were also observed, especially for length of stay for ISs (median [IQR] days, 11.5 [10.1-12.9]) and TIA (median [IQR] days, 8.1 [6.8-9.5]) (eTable 6 in the Supplement). The most notable rural vs urban differences of in-hospital management were IV-rtPA administration of 4.5 hours or less (rate, 20.2% [95% CI, 19.9%-20.5%] vs 25.9% [95% CI, 25.6%-26.1%]; absolute standardized difference, 13.6%) and anticoagulants for AF (rate, 44.3% [95% CI, 43.7%-45.0%] vs 50.7% [95% CI, 50.2%-51.3%]; absolute standardized difference, 12.8%) (eTable 7 in the Supplement). No significant differences were observed for in-hospital outcomes between urban and rural hospitals (eTable 8 in the Supplement).

## Discussion

In this quality improvement study, the adherence to performance measures varied widely by the type of cerebrovascular event. Compared with patients with IS, poor outcomes and complications were substantially higher among patients with ICH and SAH and substantially lower among patients with TIA. Substantial improvements in stroke and TIA care quality were observed from 2015 to 2019, especially in IV-rtPA administration, dysphagia screening, and administration of anticoagulants for atrial fibrillation. Improvements in in-hospital death or DAMA and in-hospital complications were also observed. However, variations in hospital management measures and in-hospital outcomes remain.

The CSCA is the largest stroke registry and quality care improvement project conducted in China to date, with more than 1 000 000 patients admitted with acute stroke or TIA. This project included stroke and TIA admissions from a large variety of hospitals from all regions in China, including a combination of secondary and tertiary as well as rural and urban hospitals.^15^ Demographic and clinical characteristics of patients with stroke or TIA enrolled in the CSCA are similar to those of other large stroke studies.^8,14,22^ Data from the Hospital Quality Monitoring System, the largest national inpatient administrative database in China, indicate that the mean age for patients with stroke is approximately 66 years and approximately 40% of patients are women.^22^ Prevalences of vascular risk factors, including prior stroke or TIA, hypertension, diabetes, and ever smoking, from other data sources were all comparable with the CSCA population.^22^ The comparability of the CSCA population with those of other large stroke studies^8,14^ supports the notion that the selection of hospitals into clinical registries may have had only minimal bias and could produce findings similar to those in studies^20,22^ with community-based cohorts or nationally representative databanks.

Few studies^20,22,23^ have assessed gaps in in-hospital management measures and outcomes by the type of cerebrovascular event. Prior reports^20,23^ from GWTG-Stroke revealed that there may be differences in performance measures across all cerebrovascular event types, although reasons behind these differences are complex. Possible explanations include variations in stroke severity or prestroke functional status, among other factors related to characteristics of patients, physicians, or hospitals. We observed that in-hospital performance measurements in the CSCA are still much lower than those reported in GWTG-Stroke 10 years ago, especially for IV-rtPA, DVT prophylaxis, and anticoagulants for AF. Because these measurements are crucial for primary treatment or secondary prevention of stroke, more targeted efforts to improve these measures in China are needed.

Similar to previous studies,^20,22^ patients with ICH and SAH in the CSCA program had higher in-hospital mortality rates than patients with IS or TIA. However, the mortality rate in the CSCA was much lower than that reported in GWTG-Stroke and clinical registries in other countries.^20^ This finding may be attributable to the exclusion of out-of-hospital deaths and emergency department deaths by design in the CSCA, in addition to advances in clinical practice over time. However, it is also likely that major contributors to the low mortality rate are culture and care affordability; many patients withdraw from treatment and leave the hospital against medical advice at the terminal stage of their disease. Taken together, these findings suggest that in-hospital death could be underestimated in China if traditional measures are used, which is consistent with a prior report^24^ from the China–Patient-Centered Evaluative Assessment of Cardiac Events project. In this analysis, to capture a more accurate estimate of mortality, we focused on a composite outcome of in-hospital death and DAMA, which may represent a unique discharge pattern in China and be more reflective of poor outcomes than in-hospital death alone. Other countries with a similar care pattern may consider using this measure of in-hospital death.

Substantial improvements over time in in-hospital management and outcome measures were observed from 2015 to 2019. The CSCA program provided a multifaceted intervention, a continuous quality improvement initiative, and a platform to develop stroke centers as well as to improve stroke care quality and clinical outcomes. These initiatives and process improvements may have also translated into the modestly improved outcomes observed. However, because most of the key process measures aim to reduce long-term disability and prevent recurrent cardiovascular events, factors other than improved process measures could have contributed to the observed improvement of acute, in-hospital outcomes. Whether the improvements in various outcomes over time in the CSCA cohort are the result of improved stroke care, national secular trends, or other factors requires further research.

### Strengths and Limitations

Our study has several major strengths. To our knowledge, the CSCA is the largest and most up-to-date quality report of patients hospitalized with acute stroke and TIA in China. With more than 1400 centers and more than 1 million patients, our study provided ample data with which to investigate important questions on the quality of care. Furthermore, the CSCA performed prospective data collection on an extensive set of variables on demographic and clinical characteristics and hospital-based performance measures, with available longitudinal data for 4 years. These methods improved our confidence in study results that indicated substantial improvements in performance measures and clinical outcomes across a diverse sample of participating hospitals.

Our study also has several limitations. Participation in the CSCA was voluntary, and the CSCA did not have an elaborately designed sampling frame. The participating hospitals were more likely to be larger teaching hospitals with a strong interest in stroke care improvement. The large sample size and a similar profile of patient characteristics to other registries may help improve the study's robustness and generalizability. In our study, we defined care quality using 11 well-accepted and predefined measures that address admission and discharge care. However, these measures do not apply uniformly to all cerebrovascular event types, and other newly advocated measures could not be calculated. In addition, some vascular risk factors (eg, prior CHD, MI, and atrial fibrillation) may have been underreported as a result of medical histories based on self-report. Another limitation was that the NIHSS score was missing for 26% of the patients, and DAMA information was missing for 23%. To mitigate and quantify the effect of missingness, we used a different level of covariates adjustment to assess the influence of missing data on the NIHSS score when assessing temporal trends; temporal trends of DAMA were evaluated from 2017 because missing data were primarily from before 2017. Additionally, data collected by hospitals were not independently audited by external medical record review. However, workshops and webinars were provided to hospitals by China Stroke Association staff and volunteers with expertise in clinical science and quality improvement, thus minimizing problems in patient enrollment and data collection.

## Conclusions

The current study described the characteristics, performance measures, and in-hospital clinical outcomes in a cohort of more than 1 million acute stroke and TIA hospitalizations from every province in China. Although substantial improvements in performance measures and in-hospital death were observed, variations by cerebrovascular event type or hospital were found, suggesting that ongoing support for evidence-based care is needed.

## Notes

Supplement. **eMethods.** Participating hospitals in CSCA **eFigure 1.** Participants and Hospitals Distribution in CSCA **eFigure 2.** Patients Enrollment Chart **eFigure 3.** Participants Enrollment in CSCA by Quarter **eTable 1.** Distribution of Participating Hospital in CSCA **eTable 2.** Specifications of Guideline-Recommended Performance Measures **eTable 3.** Trend Analysis of Quality Measurement **eTable 4.** Trend Analysis of In-Hospital Outcomes **eTable 5.** Variations of Performance Measures at Hospital Level **eTable 6.** Variations of Stroke-Related In-Hospital Outcomes and Complications at Hospital Level **eTable 7.** Performance Measures by Localization **eTable 8.** Stroke-Related In-Hospital Outcomes and Complications by Localization
