# Data Errors and a Wording Error

**DOI:** 10.1001/jamanetworkopen.2021.29006

**Published:** 2021-09-03

In the Original Investigation titled “Clinical Characteristics, Management, and In-Hospital Outcomes in Patients With Stroke or Transient Ischemic Attack in China,”^1^ published August 13, 2021, there were 2 errors in the data. In Table 3, the rate of cerebral infarction in individuals with transient ischemic attack (TIA) has been amended to 684 of 64 929 patients (1.1%; 95% CI, 1.0%-1.1%); consequently, the rate of in-hospital major adverse cardiovascular events in patients with TIA has been changed to 2024 (3.1%; 95% CI, 3.0%-3.3%) in Table 3 and in the Abstract; a new Supplement has been provided to reflect these changes. In addition, a sentence in Results has been revised to read “Variations of in-hospital outcomes were also observed, except for length of stay for ISs (median [IQR] days, 11.5 [10.1-12.9]) and TIA (median [IQR] days, 8.1 [6.8-9.5]).” These changes do not alter the authors’ conclusions. This article has been corrected.^1^
