# Supplementary material for: Clinical Characteristics, Management, and In-Hospital Outcomes in Patients With Stroke or Transient Ischemic Attack in China
Source: JAMA Netw Open. 2021 Aug 13;4(8):e2120745. doi: 10.1001/jamanetworkopen.2021.20745 (PMC8363916; doi:10.1001/jamanetworkopen.2021.20745)
Supplement: Supplement. — eMethods. Participating hospitals in CSCA eFigure 1. Participants and Hospitals Distribution in CSCA eFigure 2. Patients Enrollment Chart eFigure 3. Participants Enrollment in CSCA by Quarter eTable 1. Distribution of Participating Hospital in CSCA eTable 2. Specifications of Guideline-Recommended Performance Measures eTable 3. Trend Analysis of Quality Measurement eTable 4. Trend Analysis of In-Hospital Outcomes eTable 5. Variations of Performance Measures at Hospital Level eTable 6. Variations of Stroke-Related In-Hospital Outcomes and Complications at Hospital Level eTable 7. Performance Measures by Localization eTable 8. Stroke-Related In-Hospital Outcomes and Complications by Localization [file jamanetwopen-e2120745-s001.pdf]

Supplementary Online Content

Gu HQ, Yang X, Wang CJ, et al. Clinical characteristics, management, and in-hospital outcomes in patients with stroke or transient ischemic attack in China. *JAMA Netw Open*. 2021;4(8):e2120745. doi:10.1001/jamanetworkopen.2021.20745

eMethods. Participating Hospitals in CSCA
eFigure 1. Participants and Hospitals Distribution in CSCA
eFigure 2. Patients Enrollment Chart
eFigure 3. Participants Enrollment in CSCA by Quarter
eTable 1. Distribution of Participating Hospital in CSCA
eTable 2. Specifications of Guideline-Recommended Performance Measures
eTable 3. Trend Analysis of Quality Measurement
eTable 4. Trend Analysis of In-Hospital Outcomes
eTable 5. Variations of Performance Measures at Hospital Level
eTable 6. Variations of Stroke-Related In-Hospital Outcomes and Complications at Hospital Level
eTable 7. Performance Measures by Localization
eTable 8. Stroke-Related In-Hospital Outcomes and Complications by Localization

This supplementary material has been provided by the authors to give readers additional information about their work.

eMethods 1. Participating hospitals in CSCA

CSCA was made available to all secondary and tertiary hospitals in China. Secondary hospitals are regional hospitals with 100 to 500 beds that provide comprehensive medical care, as well as medical education and medical research on a regional basis. Secondary hospitals are usually located in a medium-sized city, county, or district. Tertiary hospitals are usually located in city centers with more than 500 beds, and they serve as medical hubs for multiple regions, with comprehensive and specialized medical care being provided. Hospitals participating in CSCA covered every province of China. Detailed distribution can be found in eFigure 1 and eTable 1.

© 2021 Gu H-Q et al. *JAMA Network Open.*

eFigure 1. Participants and hospitals distribution in CSCA

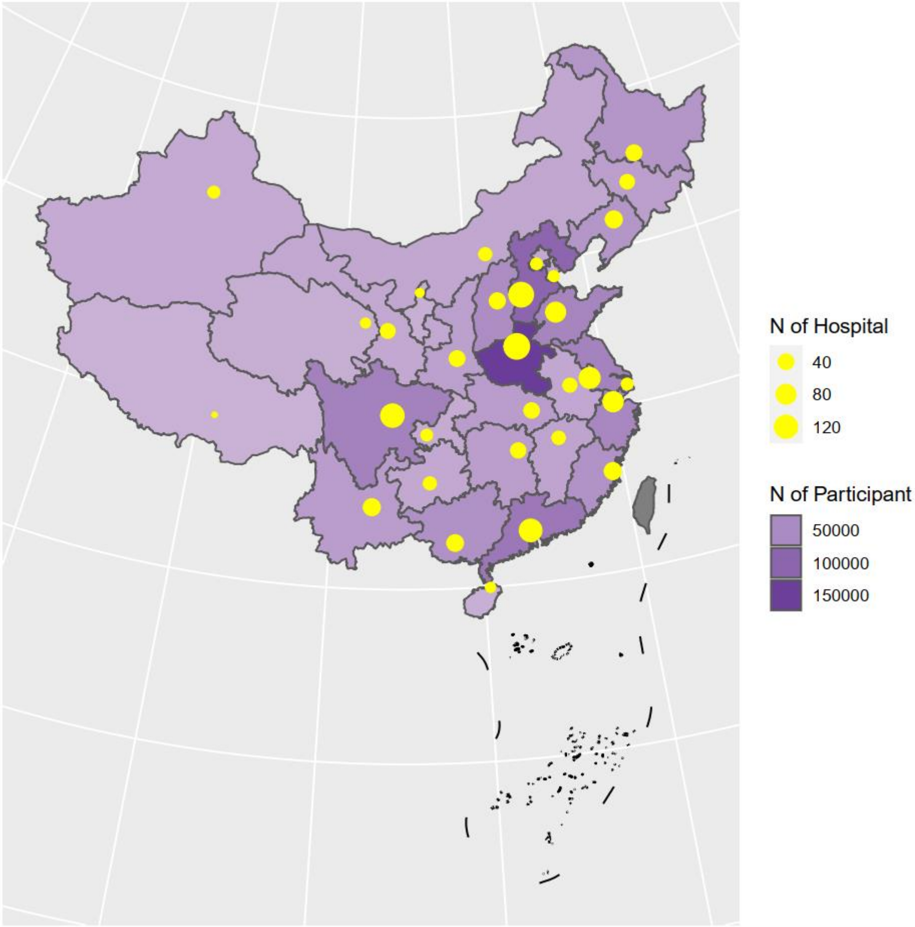

Abbreviations: CSCA indicatesThe Chinese Stroke Center Alliance.

© 2021 Gu H-Q et al. *JAMA Network Open.*

eFigure 2. Patients enrollment chart

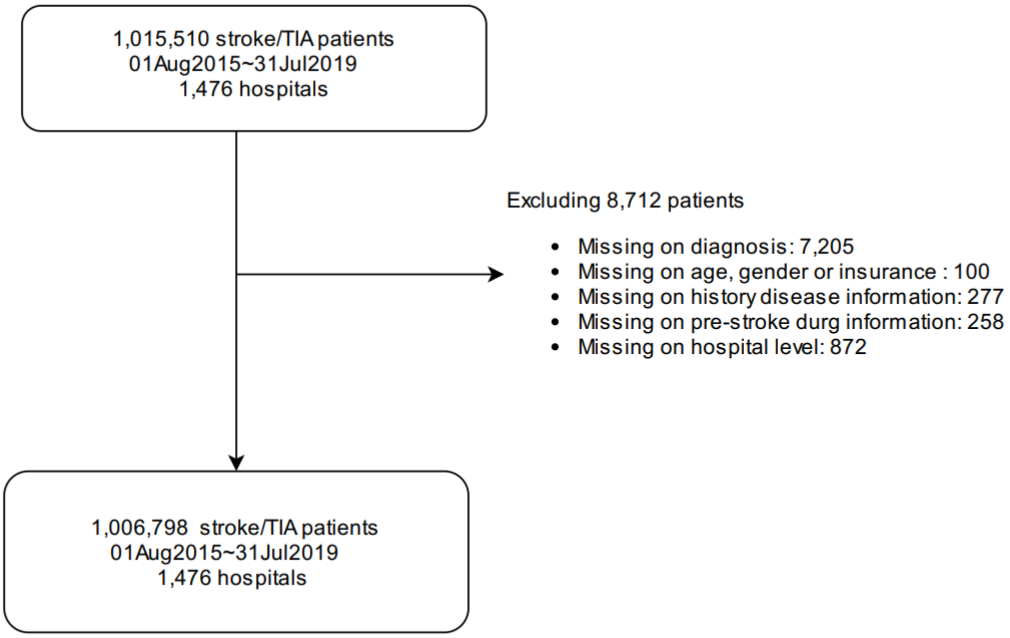

Abbreviations: TIA indicates transient ischemic stroke

© 2021 Gu H-Q et al. *JAMA Network Open.*

eFigure 3. Participants enrollment in CSCA by quarter

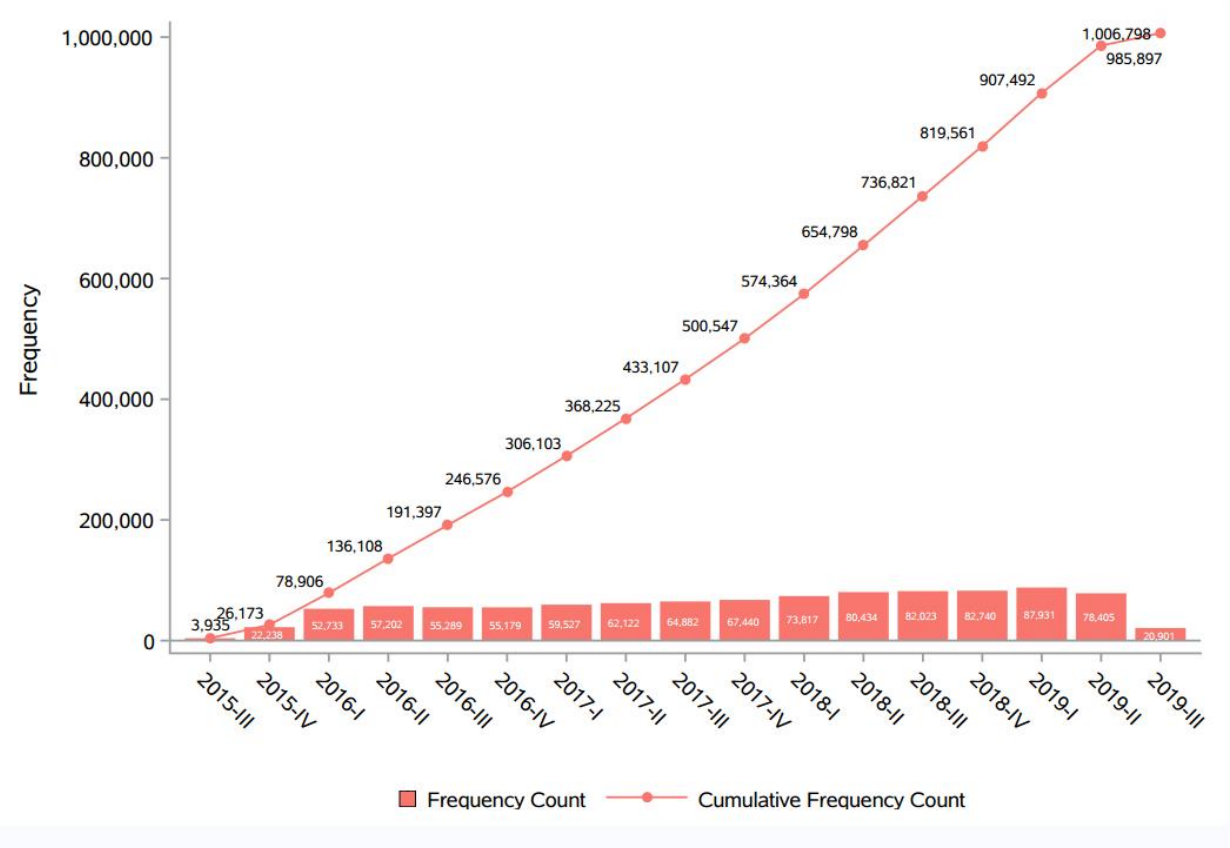

Abbreviations: CSCA indicates the Chinese Stroke Center Alliance.

© 2021 Gu H-Q et al. *JAMA Network Open.*

eTable 1. Distribution of participating hospital in CSCA

Area	n (%)* for secondary hospital	n (%)* for Tertiary hospital
Anhui	15 (4.3)	14 (20.6)
Beijing	5 (3.6)	11 (11.1)
Fujian	19 (9.7)	33 (47.1)
Gansu	20 (10.9)	14 (38.9)
Guangdong	32 (7.1)	81 (47.6)
Guangxi	19 (7.8)	30 (41.7)
Guizhou	10 (3.5)	13 (26)
Hainan	3 (7.7)	7 (41.2)
Hebei	110 (21.6)	33 (47.8)
Henan	122 (26)	35 (40.2)
Heilongjiang	14 (4.1)	27 (28.4)
Hubei	15 (4.8)	24 (19.5)
Hunan	18 (5.2)	19 (26)
Jilin	12 (5.5)	21 (45.7)
Jiangsu	39 (10.9)	54 (35.1)
Jiangxi	12 (5.3)	13 (19.7)
Liaoning	22 (7.3)	31 (24.2)
Inner Mongolia	5 (1.7)	18 (25)
Ningxia	0 (0)	5 (38.5)
Qinghai	1 (1.1)	8 (44.4)
Shandong	48 (8.1)	33 (19.9)
Shanxi	26 (7.4)	18 (31)
Shaanxi	18 (5.2)	23 (40.4)
Shanghai	7 (6.7)	9 (19.1)
Sichuan	43 (7.4)	82 (53.9)
Tianjing	6 (8.2)	7 (16.7)
Tibet	0 (0)	1 (9.1)
Xingjiang	10 (4.3)	7 (18.4)
Yunan	32 (9.4)	20 (29)
Zhejiang	28 (12.3)	58 (43.6)
Chongqing	9 (6.3)	7 (17.1)

* % was calculated based on hospital data of *China Health and Family Planning Statistics Yearbook 2018*

© 2021 Gu H-Q et al. *JAMA Network Open.*

eTable 2. Specifications of guideline-recommended performance measures

Performance measure of ischemic stroke care	Performance measure definition for eligible patients
Admission performance measures	
IV-rtPA < 4.5 Hours	Intravenous recombinant tissue plasminogen activator (IV rtPA) in patients who arrive within 3.5 hours after initial symptom onset and treated within 4.5 hours
Early antithrombotics	Antithrombotic therapy prescribed within 2 days of hospitalization, including antiplatelet or anticoagulant therapy
DVT prophylaxis	Patients at risk for deep vein thrombosis (DVT) (non-ambulatory) who received DVT prophylaxis by end of hospital day two, including pneumatic compression, warfarin sodium or heparin sodium
Dysphagia screening	Dysphagia screening prior to any oral intake
Rehabilitation assessment	Assessed for stroke rehabilitation services
Performance measures at discharge	
Antithrombotic medication	Antithrombotic therapy prescribed at discharge
Anticoagulation for atrial fibrillation	Anticoagulation prescribed at discharge for patients with atrial fibrillation or atrial flutter documented during the hospitalization
Antihypertensive medicines for patients with hypertension	Antihypertension medication prescribed at discharge for patients with history of hypertension disease or hypertension disease documented during the hospitalization
Hypoglycemia medication for diabetes	Hypoglycemic medication prescribed at discharge for patients with history of diabetes mellitus or diabetes mellitus documented during the hospitalization
Statin for lowering low-density lipoprotein ≥100 mg/dL or not documented	Statin prescribed at discharge if low-density lipoprotein (LDL) ≥100 mg/dL, if patient treated with lipid lowering agent prior to admission, or LDL not documented.

© 2021 Gu H-Q et al. *JAMA Network Open.*

Smoking cessation intervention	Smoking cessation intervention (counseling or medication) prior to discharge for current or recent smokers

Eligible patients are those without any medical contraindications (e.g., treatment intolerance, excessive risk of adverse reaction, patient/family refusal, or terminal illness/comfort care only) documented as reasons for non-treatment for each of the applicable measures. Acute performance measures, except for rt-PA measure, exclude patients who died before the end of hospital day two. Performance measures at discharge exclude patients who died during hospitalization.

© 2021 Gu H-Q et al. *JAMA Network Open.*

eTable 3. Trend analysis of quality measurement

Variables	2015 (N=26173 [2.6%])	2016 (N=220403 [21.9%])	2017 (N=253971 [25.2%])	2018 (N=319014 [31.7%])	2019 (N=187237 [18.6%])	Relative Increasement from 2015 to 2019 % (95%CI)	P_{trend}	Adjusted P_{trend}	NIHSS-Adjusted P_{trend}
Admission performance measures[a]									
IV rt-PA ≤ 4.5h	658 (17.9)	5949 (16.5)	9290 (21.0)	16270 (24.2)	11582 (28.7)	60.3 (52.9 to 70.5)	<0.001	<0.001	<0.001
Early antithrombotics	18657 (84.6)	165093 (86.2)	190324 (85.0)	241213 (84.4)	145122 (85.7)	1.3 (0.9 to 1.7)	<0.001	0.32	0.21
DVT prophylaxis	3837 (16.2)	12379 (16.9)	14439 (17.2)	18197 (18.0)	9755 (18.4)	13.6 (12.7 to 15.3)	<0.001	<0.001	<0.001
Dysphagia screen	17455 (66.7)	155507 (70.6)	177773 (70.0)	236666 (74.2)	143317 (76.5)	14.7 (14 to 15.6)	<0.001	<0.001	<0.001
Rehabilitation	17848 (68.2)	152628 (69.2)	180663 (71.1)	234963 (73.7)	135373 (72.3)	6.0 (5.4 to 6.7)	<0.001	<0.001	<0.001
Discharge Performance Measures[a]									
Antithrombotics	19122 (88.0)	168495 (89.1)	195087 (88.1)	246947 (87.3)	147997 (88.1)	0.1 (-0.1 to 0.5)	<0.001	<0.001	<0.001
Anticoagulants for AF	631 (35.4)	4910 (37.0)	6284 (40.1)	8739 (43.6)	5256 (46.5)	31.4 (25.7 to 37.3)	<0.001	<0.001	<0.001
BP lowering for HTN	13547 (68.6)	107294 (65.4)	126775 (66.5)	158471 (66.1)	92957 (65.9)	-3.9 (-4.5 to -3.5)	0.29	0.46	0.01
Glucose-lowering for hyperglycemia	5019 (77.1)	41699 (78.1)	50233 (78.3)	65008 (77.8)	38418 (78.1)	1.3 (0.5 to 2.1)	<0.001	<0.001	<0.001
Statin for LDL≥100 or ND	19909 (89.7)	174140 (90.4)	202084 (89.6)	256782 (89.1)	153961 (90.1)	0.4 (0.1 to 0.7)	<0.001	<0.001	<0.001
Smoking cessation intervention	6211 (96.4)	49819 (95.7)	55915 (96.1)	70069 (96.0)	40398 (96.3)	-0.1 (-0.5 to 0.1)	<0.001	<0.001	<0.001
Summary measures[a]									
Composite score	0.75±0.23	0.75±0.23	0.75±0.22	0.76±0.22	0.77±0.22	2.6 (2.3 to 2.8)	<0.001	<0.001	<0.001
All-or-none	7004 (26.8)	56160 (25.5)	63520 (25.0)	83618 (29.5)	51866 (27.7)	3.4 (2.2 to 5.0)	<0.001	<0.001	0.02

Abbreviations: IS indicates ischemic stroke; TIA, transient ischemic attack; ICH, intracerebral hemorrhage; SAH, subarachnoid hemorrhage; SNC, stroke not classified;

© 2021 Gu H-Q et al. *JAMA Network Open.*

IV rt-PA, Intravenous recombinant tissue plasminogen activator; DVT, deep vein thrombosis; AF, atrial fibrillation; HTN, hypertension; LDL, low-density lipoprotein; and ND, not documented.

[a] measures were assessed among eligible patients only.

© 2021 Gu H-Q et al. *JAMA Network Open.*

eTable 4. Trend analysis of in-hospital outcomes

Variables	2015 (N=26173 [2.6%])	2016 (N=220403 [21.9%])	2017 (N=253971 [25.2%])	2018 (N=319014 [31.7%])	2019 (N=187237 [18.6%])	Relative change from 2015 to 2019 % (95% CI)	P_{trend}	Adjusted P_{trend}	NIHSS-Adjusted P_{trend}
All stroke/TIA patients									
In hospital death/DAMA[a]			18259 (7.2)	21464 (6.7)	12195 (6.5)	-9.7 (-9.6 to -8.5)	<.001	<.001	<.001
In-hospital MACE	1766 (6.8)	13078 (5.9)	15366 (6.1)	21038 (6.6)	12872 (6.9)	1.5 (-1.4 to 7.8)	<.001	<.001	<.001
In-hospital Complications	4451 (17.0)	32848 (14.9)	35383 (13.9)	43082 (13.5)	23163 (12.4)	-27.1 (-28.6 to -25.3)	<.001	<.01	<.001
IS									
In hospital death/DAMA[a]			12813 (6.1)	15812 (5.9)	9161 (5.7)	-6.6 (-6.7 to -6.5)	<.0001	<.001	<.001
In-hospital MACE	1449 (7.0)	10268 (5.8)	12402 (5.9)	17918 (6.7)	11131 (7.0)	0.0 (-4.1 to 1.5)	<.001	<.001	<.001
In-hospital Complications	3255 (15.8)	24456 (13.7)	26749 (12.7)	34238 (12.7)	18723 (11.7)	-25.9 (-27 to -24.2)	<.001	<.001	<.001
TIA									
In hospital death/DAMAa			933 (5.8)	877 (4.5)	545 (4.7)	-19.0 (-20.4 to -18.0)	<.001	0.002	NA
In-hospital MACE	44 (2.4)	367 (2.3)	339 (2.1)	507 (2.6)	280 (2.4)	0 .0(-12.9 to 23.5)	0.56	0.98	0.23
In-hospital Complications	17 (0.9)	118 (0.7)	110 (0.7)	153 (0.8)	92 (0.8)	-11.1 (-28.6 to 20)	0.47	0.91	0.66
ICH									
In hospital death/DAMA[a]			3752 (16.6)	3968 (16.3)	2079 (16.1)	-3.0 (-3.8 to -1.2)	0.20	<.001	0.24

© 2021 Gu H-Q et al. *JAMA Network Open.*

In-hospital MACE	219 (6.9)	2041 (9.2)	2228 (9.8)	2241 (9.1)	1271 (9.8)	42.0 (32.1 to 55.0)	0.01	0.26	0.005
In-hospital Complications	1023 (32.4)	7198 (32.4)	7277 (31.9)	7492 (30.5)	3801 (29.3)	-9.6 (-11.5 to -7.2)	<.001	<.001	0.96
SAH									
In hospital death/DAMA[a]			660 (21.4)	703 (21.2)	323 (20.3)	-5.1 (-8.5 to -2.6)	0.35	<.001	0.01
In-hospital MACE	43 (10.2)	344 (12.3)	293 (9.5)	279 (8.3)	123 (7.7)	-24.5 (-31.3 to -12.3)	<.0001	<.001	0.09
In-hospital Complications	130 (30.7)	921 (32.9)	1041 (33.8)	991 (29.6)	448 (28.1)	-8.5 (-13.4 to -1.5)	<.001	0.003	0.57
SNC									
In hospital death/DAMA[a]			101 (5.9)	104 (4.6)	87 (7.9)	33.9 (31.3 to 35.7)	0.070	0.33	NA
In-hospital MACE	11 (8.0)	58 (4.0)	104 (6.0)	93 (4.1)	67 (6.1)	-23.8 (-40.5 to 34.3)	0.56	0.19	0.82
In-hospital Complications	26 (19.0)	155 (10.6)	206 (12.0)	208 (9.2)	99 (9.0)	-52.6 (-58 to -41.1)	0.001	0.18	0.029

Abbreviations: IS indicates ischemic stroke; TIA: transient ischemic attack; ICH: intracerebral hemorrhage; SAH: subarachnoid hemorrhage; SNC: stroke not classified; DAMA: discharge against medical advice; MACE: major adverse cardiovascular event;

[a]As DAMA were not warned when it was missing in the system before 2017; therefore, over 90% of them were missing. As thus, the trend analyses were assessed starting from 2017.

© 2021 Gu H-Q et al. *JAMA Network Open.*

eTable 5. Variations of performance measures at hospital level.

Variables	IS median (IQR)	TIA median (IQR)	ICH median (IQR)	SAH median (IQR)	SNC median (IQR)
Admission performance measures					
IV-rtPA ≤ 4.5h	16.9 (5.6–33.3)	NA	NA	NA	NA
Early antithrombotics	87.7 (76.1–93.9)	90.0 (75.0–98.2)	NA	NA	NA
DVT prophylaxis	7.7 (1.9–20.9)	NA	0.0 (0.0–21.1)	0.0 (0.0–10.0)	0.0 (0.0–0.0)
Dysphagia screen	82.8 (66.1–92.7)	NA	80.0 (57.9–93.6)	75.0 (46.7–100.0)	71.4 (28.6–100.0)
Rehabilitation	81.1 (61.3–92.8)	NA	76.3 (55.6–92.6)	55.6 (16.7–100.0)	78.0 (25.0–100.0)
Discharge performance measures					
Antithrombotics	90.1 (81.4–95.0)	92.9 (80.0–100.0)	NA	NA	NA
Anticoagulants for AF	37.5 (22.6–52.5)	33.3 (0.0–66.7)	NA	NA	NA
BP lowering for HTN	64.0 (54.9–72.5)	65.0 (50.0–77.8)	86.1 (76.9–95.0)	78.6 (50.0–100.0)	69.1 (33.3–100.0)
Glucose-lowering for hyperglycemia	79.5 (73.1–85.2)	81.8 (66.7–100.0)	75.0 (52.0–100.0)	66.7 (0.0–100.0)	92.7 (40.0–100.0)
Statin for LDL⩾100 or ND	92.4 (86.2–95.8)	94.9 (85.7–100.0)	NA	NA	NA
Smoking cessation intervention	98.1 (95.1–100.0)	100.0 (100.0–100.0)	100.0 (93.3–100.0)	100.0 (100.0–100.0)	100.0 (100.0–100.0)
Summary measures					
Compoiste score	0.8 (0.7–0.8)	0.9 (0.8–0.9)	0.7 (0.6–0.8)	0.6 (0.5–0.7)	0.6 (0.5–0.8)
All-or-none	19.4 (10.6–29.5)	54.5 (40.7–66.7)	25.0 (9.7–41.7)	8.0 (0.0–33.3)	0.0 (0.0–30.0)

Abbreviations: IS indicates ischemic stroke; TIA, transient ischemic attack; ICH, intracerebral hemorrhage; SAH, subarachnoid hemorrhage; SNC, stroke not classified; IQR, interquartile range; IV-rtPA, Intravenous recombinant tissue plasminogen activator; DVT, deep vein thrombosis; AF, atrial fibrillation; HTN, hypertension; LDL, low-density lipoprotein; ND, not documented; and NA, not applicable.

© 2021 Gu H-Q et al. *JAMA Network Open.*

eTable 6. Variations of stroke-related in-hospital outcomes and complications at hospital level.

Variables	IS<br>median (IQR)	TIA<br>median (IQR)	ICH<br>median (IQR)	SAH<br>median (IQR)	SNC<br>median (IQR)
In hospital death/DAMA	3.5 (1.2–7.0)	0.0 (0.0–5.5)	5.2 (0.0–14.3)	0.0 (0.0–25.0)	0.0 (0.0–0.0)
In-hospital MACE	5.6 (2.9–9.6)	20.0 (0.0–50.0)	6.8 (0.0–15.0)	0.0 (0.0–11.4)	0.0 (0.0–4.0)
Length of stay	11.5 (10.1–12.9)	8.1 (6.8–9.5)	14.9 (12.6–18.1)	13.2 (9.0–17.6)	9.4 (7.0–13.0)
In-hospital Complications	12.0 (7.7–17.6)	0.0 (0.0–8.3)	24.7 (13.3–38.1)	21.4 (0.0–47.3)	0.0 (0.0–25.0)

Abbreviations: IS indicates ischemic stroke; TIA, transient ischemic attack; ICH, intracerebral hemorrhage; SAH, subarachnoid hemorrhage; SNC, stroke not classified; IQR , interquartile range; DAMA, discharge against medical advice; and MACE, major adverse cardiovascular event.

© 2021 Gu H-Q et al. *JAMA Network Open.*

eTable 7. Performance measures by localization.

	Rural (N=418545 [41.6%])		Urban (N=588253 [58.4%])		
Variables	Relative frequency	% (95% CI)	Relative frequency	% (95% CI)	ASD (%)[a]
Admission performance measures					%
IV-rtPA ≤ 4.5h	16153/80032	20.2 (19.9–20.5)	27845/107606	25.9 (25.6–26.1)	13.6
Early antithrombotics	316546/366052	86.5 (86.4–86.6)	444419/520855	85.3 (85.2–85.4)	3.4
DVT prophylaxis	24648/127925	19.3 (19.1–19.5)	33959/192418	17.7 (17.5–17.8)	4.1
Dysphagia screen	300558/389491	77.2 (77.0–77.3)	430160/548711	78.4 (78.3–78.5)	2.9
Rehabilitation	299553/418420	71.6 (71.5–71.7)	421922/587959	71.8 (71.7–71.9)	0.4
Discharge performance measures					
Antithrombotics	324714/379517	85.6 (85.5–85.7)	456664/539158	84.7 (84.6–84.8)	2.5
Anticoagulants for AF	8773/19787	44.3 (43.7–45.0)	17206/33917	50.7 (50.2–51.3)	12.8
BP lowering for HTN	205093/256054	80.1 (79.9–80.3)	293951/363062	81.0 (80.8–81.1)	2.3
Glucose-lowering for hyperglycemia	72860/83714	87.0 (86.8–87.3)	127517/147256	86.6 (86.4–86.8)	1.2
Statin for LDL≥100 or ND	346633/408296	84.9 (84.8–85.0)	482278/573913	84.0 (83.9–84.1)	2.5
Smoking cessation intervention	81904/84870	96.5 (96.4–96.6)	140508/146192	96.1 (96.0–96.2)	2.1
Summary measures					
Composite score		0.76±0.22		0.75±0.22	4.5
All-or-none	111588/418540	26.7 (26.5–26.8)	150580/588226	25.6 (25.5–25.7)	2.5

Abbreviations: IS indicates ischemic stroke; TIA, transient ischemic attack; ICH, intracerebral hemorrhage; SAH, subarachnoid hemorrhage; SNC, stroke not classified; IV-rtPA, Intravenous recombinant tissue plasminogen activator; DVT, deep vein thrombosis; AF, atrial fibrillation; HTN, hypertension; LDL, low-density lipoprotein; ND, not documented; ASD, absolute standard difference.

[a]an ASD of greater than ten is considered clinically significant.

© 2021 Gu H-Q et al. *JAMA Network Open.*

eTable 8. Stroke-related in-hospital outcomes and complications by localization.

	Rural (N=418545 [41.6%])		Urban (N=588253 [58.4%])		
**Variables**	Relative frequency	% (95% CI)	Relative frequency	% (95% CI)	ASD (%)[a]
In hospital death/DAMA	2 5018/322784	7.8 (7.7–7.8)	29677/450681	6.6 (6.5–6.7)	4.6
In-hospital death	1562/418545	0.4 (0.4–0.4)	4616/588253	0.8 (0.8–0.8)	5.2
DAMA	23456/321222	7.3 (7.2–7.4)	25061/446065	5.6 (5.6–5.7)	6.9
In-hospital CVD events	26007/418545	6.2 (6.1–6.3)	38113/588253	6.5 (6.4–6.5)	1.2
Cerebral infarction	17299/418545	4.1 (4.1–4.2)	26298/588253	4.5 (4.4–4.5)	2.0
Cerebral hemorrhage	6566/418545	1.6 (1.5–1.6)	9177/588253	1.6 (1.5–1.6)	0.0
TIA	3385/418545	0.8 (0.8–0.8)	4587/588253	0.8 (0.8–0.8)	0.0
Myocardial infarction	1357/418545	0.3 (0.3–0.3)	2400/588253	0.4 (0.4–0.4)	1.7
In-hospital Complications	53582/418545	12.8 (12.7–12.9)	85345/588253	14.5 (14.4–14.6)	5.0
DVT	3280/418545	0.8 (0.8–0.8)	5832/588253	1.0 (1.0–1.0)	2.1
Pneumonia	38953/418545	9.3 (9.2–9.4)	63014/588253	10.7 (10.6–10.8)	4.7
Pulmonary embolism	736/418545	0.2 (0.2–0.2)	1324/588253	0.2 (0.2–0.2)	0.0
Epileptic Seizure	2402/418545	0.6 (0.6–0.6)	3841/588253	0.7 (0.6–0.7)	1.2
Hydrocephalus	1462/418545	0.4 (0.3–0.4)	2522/588253	0.4 (0.4–0.5)	0.0
Urinary infection	4628/418545	1.1 (1.1–1.1)	8642/588253	1.5 (1.4–1.5)	3.5
Respiratory failure or cardiopulmonary arrest	1561/418545	0.4 (0.4–0.4)	3543/588253	0.6 (0.6–0.6)	2.8
Bedsore	1286/418545	0.3 (0.3–0.3)	2055/588253	0.4 (0.3–0.4)	1.7
Depression	5233/418545	1.3 (1.2–1.3)	7312/588253	1.2 (1.2–1.3)	0.9
Gastrointestinal bleeding	3872/418545	0.9 (0.9–1.0)	6885/588253	1.2 (1.1–1.2)	2.9

Abbreviations: DAMA, discharge against medical advice; and MACE, major adverse cardiovascular event, and DVT, deep vein thrombosis.

[a] absolute standard difference , an ASD of greater than ten is considered clinically significant.

© 2021 Gu H-Q et al. *JAMA Network Open.*
